# From Threatening Chaos to Temporary Order through a Complex Process of Adaptation: A Grounded Theory Study of the Escalation of Intensive Care during the COVID-19 Pandemic

**DOI:** 10.3390/ijerph20217019

**Published:** 2023-11-03

**Authors:** Camilla Göras, Malin Lohela-Karlsson, Markus Castegren, Emelie Condén Mellgren, Mirjam Ekstedt, Petronella Bjurling-Sjöberg

**Affiliations:** 1 Department of Caring Sciences, Faculty of Health and Occupational Sciences, University of Gävle, SE-801 76 Gävle, Sweden; camilla.goras@hig.se; 2 Department of Anesthesia and Intensive Care, Falu Hospital, SE-791 31 Falun, Sweden; 3 Department of Public Health and Caring Sciences, Health Services Research, Uppsala University, SE-752 37 Uppsala, Sweden; malin.lohela.karlsson@regionvastmanland.se; 4 Centre for Clinical Research Västmanland, Uppsala University, SE-721 89 Västerås, Sweden; emelie.conden.mellgren@regionvastmanland.se; 5 Department of Clinical Science, Intervention and Technology, Karolinska Institutet, SE-171 77 Stockholm, Sweden; markus.castegren@regionsormland.se; 6 Centre for Clinical Research Sörmland, Uppsala University, SE-631 88 Eskilstuna, Sweden; 7 Department of Health and Caring Sciences, Linnaeus University Kalmar/Växjö, SE-392 31 Kalmar, Sweden; mirjam.ekstedt@lnu.se; 8 Department of Learning, Informatics, Management and Ethics, Karolinska Institutet, SE-171 77 Stockholm, Sweden; 9 Department of Public Health and Caring Sciences, Caring Science, Uppsala University, SE-752 37 Uppsala, Sweden

**Keywords:** adaptive capacity, COVID-19, grounded theory, healthcare, intensive care, resilience, resilient performance, surge response, unexpected crises

## Abstract

To ensure high-quality care, operationalize resilience and fill the knowledge gap regarding how to improve the prerequisites for resilient performance, it is necessary to understand how adaptive capacity unfolds in practice. The main aim of this research was to explain the escalation process of intensive care during the first wave of the pandemic from a microlevel perspective, including expressions of resilient performance, intervening conditions at the micro-meso-macrolevels and short- and long-term consequences. A secondary aim was to provide recommendations regarding how to optimize the prerequisites for resilient performance in intensive care. A grounded theory methodology was used. First-person stories from different healthcare professionals (n70) in two Swedish regions were analyzed using the constant comparative method. This resulted in a novel conceptual model (including 6 main categories and 24 subcategories), and 41 recommendations. The conclusion of these findings is that the escalation of intensive care can be conceptualized as a transition from threatening chaos to temporary order through a complex process of adaptation. To prepare for the future, the components of space, stuff, staff, system and science, with associated continuity plans, must be implemented, anchored and communicated to actors at all levels of the system.

## 1. Introduction

To promote quality of care and patient safety, healthcare systems need to have the capacity to exhibit resilient performance in both everyday practice and major crises, such as a pandemic. Operationalizing resilience has been on the agenda for decades. However, the elucidation of ways of improving the prerequisites for resilient performance is still a work in progress [1,2]. The Coronavirus disease 2019 (COVID-19) pandemic severely tested healthcare systems, challenging their capacity to handle a surge of critically ill patients [1,3,4,5]. This included a struggle with limited initial knowledge and resources. Intensive care units (ICUs) were particularly stretched, needing a rapid and extreme increase in bed capacity [6,7]. Analyzing the ICU response during the pandemic can offer valuable insights for crafting strategies to improve healthcare resilience.

Resilience in healthcare can be defined as ‘the capacity to adapt to challenges and changes at different levels of the system to maintain high-quality care’ [2], a definition which also supports our understanding of resilience as a phenomenon. The healthcare context can be invoked as a complex adaptive system (CAS), and as such, it constantly interacts with the environment and includes many different actors and interacting systems at different levels [8,9]. One characteristic commonly attributed to a CAS is its self-organizing nature, which enables the system to remain stable despite challenges. However, changing dynamics and disruptions also have the potential to destabilize the system [10,11,12]. Healthcare is also characterized by the tensions between demand and capacity, and between work-as-imagined and work-as-done [13]. Individuals and teams within a CAS react, communicate, adapt, learn and self-organize over time [10]. Different types of adaptation to overcome misalignments are performed at different temporal and spatial scales [13,14], e.g., at different levels of the system and at different times. However, such adaptations are not normative, as their outcome can be unintended and can vary across the system and across time [13,15,16]. For example, the microlevel, such as that of a care department, sometimes operates at different temporalities than the mesolevel (such as a healthcare organization) or the macrolevel (i.e., national and international society), thus raising challenges for the ability to anticipate and understand a larger view of the system [8]. Expedient adaptations are dependent on the adaptive capacity of the organization, but the individual’s adaptations in the context of daily work are not always equivalent to building adaptive capacity within the system itself [16,17]. To understand how adaptations can support resilient performance at the micro-meso-macrolevels, consideration of the complexity of the healthcare system is suggested [9,16]. 

Intensive care is a complex technology and resource-intense operation that requires high staff density as well as specialist competencies ranging across several professions [18,19]. Prepandemic ICU capacity, the pandemic surge response and the outcomes of intensive care patients varied widely across countries and regions [5,6,7]. Internationally, during the pandemic, deficits in staff and material resources as well as a higher variation in standards of care delivery in the ICU were reported [5,6,19]. During the first wave of the pandemic, Sweden chose a different disease prevention and control path than many other European countries. The actions taken, and the key actors who were involved, are described in a detailed timeline in a paper by Ludvigsson [20], but can be summarized as no general lockdown, no enforced quarantines for infected households or geographical regions, no recommendation of facemasks outside healthcare, and a focus on slowing, not stopping, the pandemic. However, physical distancing was strongly recommended (although only mandatory in some public places) and, despite the fact that the recommendations were mainly voluntary, opinion polls revealed high compliance [20]. From March to April 2020, the growing number of patients with COVID-19 put enormous pressure on the Swedish healthcare system. Overall, Swedish ICU admissions more than doubled during the first wave of the pandemic [7], and some regions witnessed up to a fivefold increase [21]; in addition, Sweden reported slightly lower ICU mortality rates than many other countries [7]. However, staff reported a strained situation [22], patients were exposed to adverse events [23], and the effects of the pandemic are still being explored. The pandemic has led to a plethora of publications regarding what was done to meet the surge of critically ill patients. However, our understanding of how the escalating process was achieved is still insufficiently detailed. To prepare healthcare more effectively for both future crises and everyday practice, further understanding of resilient performance in ICUs is necessary [5,24].

Resilient performance requires the potential to anticipate, monitor, respond and learn [8]. In terms of surge capacity in times of a mass event such as a pandemic or other disasters, resilience is largely related to the prerequisites for the adaptive capacity of various system components, which are commonly referred to as the four Ss (space, stuff, staff and system) [24,25]. Reflecting on the need for knowledge during the current pandemic, Salluh et al. [24] suggested adding a fifth S to represent science. However, resilient healthcare is an emerging research area, and to improve our understanding of this topic and promote theoretical development in this field, a contextual understanding based on empirical data is necessary [26,27,28]. A dynamic situation such as the pandemic [3] highlights the need for a resilient healthcare system [1]. As our knowledge of how adaptive capacity can be unfolded in practice exhibits certain gaps, exploration of the underlying dynamics of work-as-done that were operative during the pandemic is of interest and importance for both academics and the healthcare community [2]. Given the unprecedented escalation in capacity, intensive care during the pandemic provides an excellent study context to explore resilient performance at different levels of the system.

The main aim of this study was to explain the escalation process of intensive care during the first wave of the pandemic from a microlevel perspective, including expressions of resilient performance, intervening conditions at the micro-meso-macrolevels and short- and long-term consequences. A secondary aim was to provide recommendations for the different levels of the system regarding how to optimize the prerequisites for resilient performance. This research is expected to contribute to the tasks of operationalizing resilience and enhancing resilient performance in both everyday practice and future crises in ICUs and other healthcare settings.

## 2. Methods 

This study was part of the multilevel research project ‘Resilient performance in healthcare during the COVID-19 pandemic (ResCOV)’, which started in 2020 and aimed to improve our understanding of the processes involved in the healthcare response to the pandemic’s rampage; for more detailed information, see the study protocol [29]. An emerging explorative design based on the grounded theory methodology [30] was used.

### 2.1. Study Context

Intensive care in two Swedish regions provided the setting for the study (Region A and Region B). Sweden, with ~10 million inhabitants, features a healthcare system that is mainly publicly funded and is managed by 21 self-governed regional councils. Despite some national regulations, each region is responsible for the management organization and prioritization of healthcare resources. The regions included in this study were of specific interest due to the exceptional burden they faced due to COVID-19 during the initial wave of the pandemic [21] and their average size nationally with a population of ~300,000. 

Before the pandemic, the total number of in-hospital beds in the study settings was ~190 beds/100,000 inhabitants, with 70–300 beds/hospital, which is a common size for local or county hospitals in Sweden. Intensive care was provided by two hospitals in each region. Criteria for admission to the ICU included patients who needed advanced care either with or without invasive mechanical ventilation. Prior to the COVID pandemic, the ICU capacity of Region A was 11 beds and that of Region B was 13 beds, with 4–8 beds/ICU (~4 beds/100,000 inhabitants), and care was provided for 1 to 4 patients per room.

**Figure 1:**
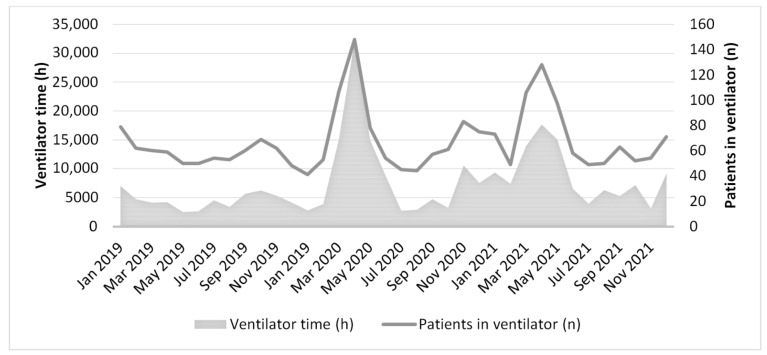
Overview of the number (n) of patients treated with invasive mechanical ventilation, as well as the ventilator hours (h), in the two studied regions from 2019 to 2021. Data is presented as the total number of patients and the total ventilator time per month. Source: Swedish ICU register [21].

During the pandemic, ICU capacity was escalated. At the peak of the first wave of contagion, in April 2020, 54 and 28 patients in Regions A and B, respectively, were simultaneously cared for in the ICUs (~18 and 9/100,000 inhabitants, respectively), which entailed increases of 491% and 215% in ICU capacity, respectively [21]. Compared to ordinary circumstances, invasive mechanical ventilation increased significantly (see Figure 1). In the most burdened region, i.e., Region A, the increase at the peak was 1080% [21]. In this region, patients with COVID-19 who were in need of intensive care received such care in the two hospitals that normally provided intensive care. In Region B, patients with COVID-19 were concentrated in only one hospital, thereby reserving intensive care in the second hospital for noncontagious patients.

During ordinary circumstances, the staff in Swedish intensive care mainly include registered nurses who are specialized in intensive care, undergraduate assistant nurses, anesthesiologists (physicians who are specialized in anesthesiology and sometimes in intensive care) and some residents (physicians receiving specialist training). This team of professionals manages all of the care for the patient, including the provision of pharmaceuticals and mechanical ventilation. Allied healthcare professions, such as physiotherapists, occupational therapists, dieticians and counselors, and other physicians are available as consultants [18]. During the pandemic, additional temporary staff drawn from other professions were engaged.

Throughout this paper, the term staff refers to all categories of healthcare professionals, including managers at different levels in the departments that managed the ICUs. When a particular professional group or management level is referenced, it is specified. The microlevel refers to the ICUs, managers and front-line staff that constitute the ICU microsystem context, i.e., both the organization and the associated individuals. The mesolevel refers to higher-level managers and other care and service departments in the regions’ healthcare systems. The macrolevel refers to the larger society and the national and international context.

### 2.2. Participants and Data Collection

Recruitment for participants in the ResCOV project started in September 2020, using an open sampling approach [30]; the goal was to gather rich data with a maximum variation from staff who were in service during the pandemic. Subsequently, guided by the simultaneously performed analysis, theoretical sampling [30] was performed with the goal of enriching the emerging findings. Information concerning the study’s aim, procedure and voluntary nature was sent broadly to the staff through the participating healthcare organizations’ e-mail systems. Individuals who volunteered to participate completed a questionnaire that collected their demographic data, and could choose either to relate their experiences in writing or to participate in an individual qualitative interview.

To facilitate the written narratives, a study-specific guide was utilized. The guide included some guiding questions pertaining to the topics of specific interest (working conditions, ethics, patient safety, adaptations, influencing factors, consequences and lessons learned) but urged the participants to communicate or omit whatever information they wanted. The interviews started with an open question: ‘Can you please tell me how the pandemic was?’ They continued with clarifying and probing questions. A semistructured interview guide, including the same topics as those included in the guide for the written narratives, was used in a flexible manner to ensure that incidents and issues pertinent to the participant were captured.

The participants in the present study represented a purposive sample drawn from the ResCOV project, consisting of 70 healthcare professionals (Table 1) who served on a regular (52.9%) or temporary (47.1%) basis in intensive care in the included regions (60% from Region A and 40% from Region B). Diversity was observed in terms of profession (52.9% registered nurses, 22.8% physicians, 15.7% managers and 8.6% assistant nurses), gender (70% women and 30% men), age (28–69 years) and experience with intensive care (ranging from zero to more than 30 years). The sample of registered nurses included nurses who were specialized in intensive care and nurses with other or no specialist training. The sample of physicians included anesthesiologists, residents and interns. The managers included both first-line (heads of care units) and second-line managers (heads of departments) of the departments that operated the ICUs (microlevel managers).

Data included the participants’ first-person stories, which were collected shortly after the first wave, i.e., from September to November 2020. Experiences were provided in the form of written narratives (67.1%) and interviews (32.9%). The interviews were performed by CG or PBS in a time and place chosen by the participants, mainly during their working time in a quiet room in the hospital or at a digital meeting. Each interview lasted for 45–120 min and was digitally recorded and transcribed verbatim.

### 2.3. Data Management and Analysis

Demographic data were processed in terms of descriptive statistics using Excel 2007 software (Microsoft, Redmond, WA, USA). Qualitative data from the participants’ stories were managed using NVivo software, release 1.3 (QRS International, Ruggell, Liechtenstein) and analyzed inductively using constant comparative analysis, including open, axial and selective coding [30]. In line with Grounded Theory [30], the analysis process started simultaneously with the data collection and continued until theoretical saturation was achieved. The analysis entailed the dynamic and iterative comparison of data, theoretical reflection, writing memos and drafting models of interpretations and associations of the emerging concepts and categories. 

To address the main aim and inductively identify concepts related to the escalation process, open coding was performed. The concepts were compared, categorized and recategorized. To identify the interrelations among the categories, axial coding was employed. The participants’ main concerns, processes and context levels were identified. A core category gradually emerged, to which the other categories were related and integrated into a conceptual model through the selective coding process. The model was validated by examining how the original empirical raw data fit and was explained by the model. Finally, a storyline was written, and some particularly illustrative quotations from the participants were selected to facilitate understanding of the phenomenon.

To address the second aim, recommendations for how to optimize the prerequisites for resilient performance were identified and conceptualized inductively, using open coding [30]. Thereafter, to facilitate practical application and possibility to relate the findings to other surge capacity literature, emerged recommendations were deductively categorized based on the five Ss (space, stuff, staff, system and science) [24,25].

Strategies appropriate for performing and reporting qualitative studies were used to ensure trustworthiness [31,32]. The primary analysis was conducted jointly by the authors who conducted the data collection, whose clinical and academic experience in the field of research ensured a theoretical sensibility. Thereafter, reflective discussions within the research group were performed throughout the analytical process. Credibility was enhanced through the collaboration of an interdisciplinary research group containing members with extensive experience in qualitative research and different professions, and both with and without experience in providing care during the pandemic.

### 2.4. Ethical Considerations

This project was approved by the Swedish Ethical Review Authority (Ref. No. 2020-04187). The voluntary nature of participation and the possibility of withdrawing without consequences were emphasized in the information letter as well as in the interviews. Regardless of participation, occupational health services were made available to any individual who experienced emotional discomfort. All data were handled in accordance with the General Data Protection Regulation (EU 2016/679) and stored securely.

## 3. Results

**Figure 2:**
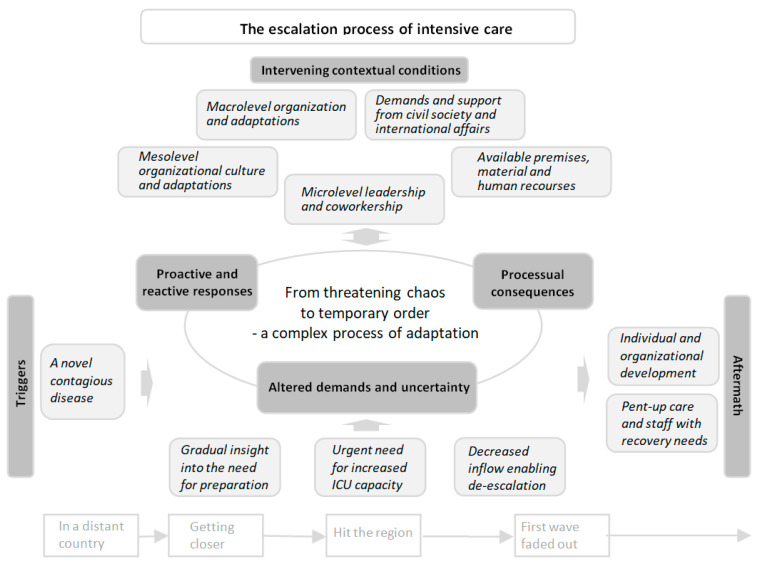
Conceptual model of the escalation process of intensive care during the first wave of the COVID-19 pandemic (the EPIC model). Visualization of a timeline extending from the appearance of the new disease until the conclusion of the first wave of the pandemic. The core category is displayed in the middle, and the main categories are displayed in bold text. Subcategories to the triggers, intervening contextual conditions and the aftermath are displayed in italics in light gray squares. Subcategories related to the other main categories are displayed in Table 2 and the storyline.

Based on the process of analyzing the participants’ stories, an overall conceptual model of the escalation process of intensive care (EPIC) emerged (see Figure 2). The model, which related to the main aim of the study, is based on 6 interconnected main categories, with in total 24 subcategories (see Table 2). A central phenomenon, which is conceptualized as *from threatening chaos to temporary order through a complex process of adaptation*, is the core category that links all categories. Related to the second aim, to optimize the prerequisites for resilient performance, 41 recommendations for different system levels emerged (see Section 3.7).

Below, all main categories of the EPIC model are presented as Section 3.1, Section 3.2, Section 3.3, Section 3.4, Section 3.5 and Section 3.6, with subcategories written in ***bold*** ***italics*** in the text, although they are occasionally written in abbreviated or grammatically modified form to facilitate reading. 

The EPIC model (Figure 2) is visualized in a timeline extending from the emergence of the new disease until the first wave of contagion faded away. The *triggers* of the escalation process created *altered demands and uncertainty*, which emerged as the main concern and generated *proactive and reactive responses*. The response led to *processual consequences* that further altered demands and forced additional responses in an iterative process of adaptation. All categories in the model were interrelated and affected by *intervening contextual conditions* at different levels of the system. The process of adaptation continued until the contagion faded away and the *aftermath* was discernible, which facilitated a successive transition *from threatening chaos to temporary order*. This transition is exemplified by the following extract from an assistant nurse’s narrative:

*“The first 4 weeks were chaos and a war zone; it was very noisy, and you had no control… Every day, every hour, you thought—I hope nothing goes wrong; I hope no patient dies on my shift… We learned a lot during this time, to be innovative and find different solutions; we were all like MacGyver! (#65).”*

### 3.1. Triggers

Overall, the ***novelty of the contagious disease*** entailed that the relevant actors lacked experience and scientific evidence regarding its transmission, treatment and prognosis. This situation led to uncertainty regarding infection-control measures and appropriate care strategies, as well as difficulty in understanding the threat and the extent of the situation. Initially, the disease was delimited and far away; it was thus perceived as ‘of no concern to us’. When reports of cases were drawing closer, the mental state of the staff shifted from ‘if’ to ‘when’ a patient with COVID-19 would arrive in the setting. This situation led to ***gradual insights*** ***into the need for preparation***, which started to alter the demands on the ICU and the individuals involved, as expressed by one interviewed physician:

*“There was news about COVID-19, and we started to wake up a little when we saw that it could affect Sweden as well. At first it was pretty obvious that this was probably not as dangerous as it looked. Then, it quite quickly changed to “this could really happen to us”. Then, it happened very quickly and it hit us. We escalated intensive care at the last minute, I would say (#28).”*

The gravity of the situation was initially underestimated, and calculations regarding the disease’s progress, including its potential effects on healthcare, were soon surpassed, when the pandemic arrived in the regions substantially earlier and with greater severity than predicted. Additionally, the patients needed more advanced care than anticipated. Hence, an ***urgent need*** ***for increased ICU capacity*** arose, which led to significantly altered demands. Eventually, after several intense months, the ***inflow decreased, and de-escalation was enabled,*** which once again altered the demands faced by the ICU organization and the individuals involved.

### 3.2. Altered Demands and Uncertainty

Altered demands and uncertainty were apparent throughout the escalation process but was most significant when the urgent need for increased ICU capacity occurred, which is exemplified by the following statement from a registered nurse:

*“I remember that everything happened at a breakneck speed, and no day was the same. Every day, there were new directives, new solutions, new personnel, new devices, new machines and new patients (#52).”*

***Rapidly altered care conditions*** were related to the rapidly altered patient inflow and frequently altered care recommendations based on gradually obtained insights into the disease. Additionally, adaptations performed in response to the altered demands further affected the conditions at the front lines. Alongside the circumstances described in all other subcategories, these factors were perceived to increase strain and to impede the ability to manage intensive care in the ordinary manner, although the surge of patients with the same diagnosis and the decreasing inflow of other patients facilitated the standardization of care and the introduction of temporary staff.

***Extensive need for room and infection control*** were related to the increased quantity of contagious patients, which required rapid diagnosis, personal protective equipment (PPE) and isolated premises to an extent that had not previously been experienced. This situation led to challenges, since the test capacity for COVID-19 was delimited, the availability of and experience with PPE was scant and ordinary ICU premises were too small for large-scale capacity escalation.

***Demand*–*capacity imbalance regarding material and human resources*** was related to this increased need and scant availability. The imbalance led to threatening and actual deficiencies in medical technology equipment (beds and mattresses, mechanical ventilators, inhalators, oxygen dispensers, airway suction devices, infusion pumps, vital function monitors, etc.), consumables (material for mechanical ventilators and infusion pumps, syringes and needles, bed linen and diapers, PPE, etc.) and pharmaceuticals (sedatives, inotropes, nutrition, etc.). Specialist competencies related to essential professions were not sufficiently available to meet the staff density requirements for intensive care. The emergency entailed that the ordinary—rigorous and slow—process of recruitment was insufficient, and there was no time for the ordinary introduction of temporary staff, which further increased the demands on ordinary staff. Additionally, the utilization of unfamiliar equipment, consumables, pharmaceuticals and PPE led to uncertainty. Subsequently, when the inflow of patients with COVID-19 decreased, staff capacity exceeded demand, which also generated frustration.

***Increased demands on governance, collaboration and communication*** were related to operational commitment and the challenges described above. The operational commitment was aimed at providing intensive care to all patients in need of this level of care, including during the pandemic. This task entailed an increased need for an overview, rapid decision-making, planning and the coordination of resources in collaboration with the whole healthcare organization. Frequent alterations and increased integration within the ICU organization and across different levels of the system, as well as the strain on front-line patient care, entailed an increased need for information dissemination and staff support. High information flow, a lack of time and limited access to computers hindered front-line information uptake. Hence, the regular management structure and regular communication and support structure were insufficient.

### 3.3. Proactive and Reactive Responses

The proactive and reactive responses included adaptations that were continually made both at the ICU management level and in front-line patient care throughout the whole escalation process. This situation was explained by one interviewed physician who served in ICU crisis management as follows:

*“We had to lay the rails while driving, and it turned out well. As good as it gets when you do that, of course. But there were a lot of decisions that were very ad hoc. It was just like this, “okay, now we’ll do this,” “well, no, we’ll do it like this instead,” and we barely had time to keep a log of all the changes we made (#38).”*

***Preparing and planning to the best of one’s ability*** was indicated by some structural adaptations that were proactively coordinated. For example, prepandemic measures were taken by individuals as well as the ICU organization to prepare for eventual cases of COVID-19 in the regions, including escalation/de-escalation plans based on different scenarios. However, due to altered demands, it was necessary to adjust plans continuously, and a large proportion of the adaptations were reactive, ad hoc solutions driven by an immediate need. Additionally, the initially rapid progress of the situation entailed limited foresight with regard to planned adaptations, sometimes on an hourly basis. As time progressed, insights and experience increased, and more proactive adaptations became possible, especially during de-escalation.

***Reorganizing the management and information structure*** was performed as a response to the altered demands on governance, collaboration and communication. An ICU crisis management group was established, which performed needs- and capacity-planning, monitored the current situation and tried to compensate for deficiencies. However, it was inherently required to establish priorities regarding how to use existing human and material resources. The ICU management also initiated actions at a higher level of the system, guiding decisions at the mesolevel, facilitating collaboration among departments, and the overall prioritization and redistribution of resources within the region and to some extent also nationally.

A structure for seeking and compiling up-to-date knowledge, and strategies for disseminating information to front-line staff through e-mail newsletters, daily meetings and strategically placed updated notes, were developed. This information included the progress of the pandemic, reorganizations and new directions and guidelines. Additionally, a considerable amount of information was disseminated among staff members informally by the “jungle drum”.

***Prioritizing and reorganizing patient flow*** was performed to mitigate the strain on ICUs and concentrate the available resources to address the most urgent need. Resource availability for intensive care was increased by deprioritizing nonimperative care and other activities. Rapid computed tomography scans for possible COVID-19 cases were organized to speed up diagnosis. Admission to the ICU was restricted to patients who were in need of invasive mechanical ventilation, and standardized criteria, including scores for fragility and comorbidity, were developed to determine whether the individual patient would benefit from such treatment. When patient inflow peaked, ICUs in other regions were utilized. In one region, an operational leadership level was established to coordinate around-the-clock patient flow.

***Restructuring and compensating premises and material resources*** started during preparation and continued throughout the escalation process. Eligible premises were adapted to meet the needs of infection control, and temporary ICUs were established in large premises that were normally used for surgery and postoperative care. Equipment, consumables and pharmaceuticals were redistributed from other parts of the healthcare organization and coordinated by designated staff. Emergency procurement was performed, and substitute equipment, trademarks and treatments were utilized. For example, simple beds without pressure-relieving functions, outmoded mechanical ventilators and ventilators that were made only for short-term anesthesia were utilized. The lack of injection pumps for precise individualized distribution and the low availability of regular sedatives entailed that the ordinary regime was substituted with pharmaceuticals that did not require pumps. The staff also prioritized patients in terms of the urgency of their need for advanced equipment and stretched the margins of safety for consumables by extending use time and reusing disposables. Additionally, PPE trademarks without regular quality certificates were used.

***Redistributing staff and adjusting roles*** were responses to demand–capacity imbalances in terms of human resources. The first response was utilizing overtime and adapting the working schedules. When this approach turned out to be insufficient, staff from other parts of the organization were redistributed to the ICU. Additionally, rental staff and staff from other regions were engaged. The urgency of the situation entailed that staffing was prioritized over qualitative introduction, and ordinary recruitment procedures and competence requirements were bypassed. To compensate for deficiencies in terms of ICU competence, the staff–patient ratio was increased. Unlike the ordinary organization, according to which physicians were on call during evenings and nights, physicians were allocated to the ICU around the clock, and their role was restricted to inside affairs. Task-shifting was performed by engaging external assisting staff (physicians in training, counselors, pharmacists, other healthcare professionals and janitors, etc.) to handle contact with the patients’ families, keep patient records, handle administration and distribute supplies.

The roles and responsibilities of ordinary staff were extended to include caring for several patients, coordinating care and simultaneously supervising temporary staff. The lack of a proper introduction was compensated for by the practice of “learning by doing”, by collaboration and collegial support when utilizing unfamiliar equipment, pharmaceuticals and PPE, by consciously making trade-offs regarding safety measures and by taking into account risks with the goal of providing care.

***Alterations and trade-offs in patient care*** were a response to the conditions on the front lines. Limited knowledge of COVID-19 was managed by drawing on evidence concerning similar diseases. Due to infection control and integrity issues in the large temporary ICUs, visiting restrictions were implemented. To increase efficiency, care in the ICU was reorganized to be more propulsive, with tests and treatments being performed around the clock regardless of night rest for the patients. Procedures, routines and treatments were streamlined and standardized. Staff counteracted chaos by planning and coordinating activities, and established priorities to distribute resources to the patients with the most urgent need. As described by a statement made by a registered nurse,

*“There were a lot of changes in my way of working that I, as an ICU nurse, had to make or completely let go of to be able to handle the situation. … I had the feeling that patient safety was lower than in the case of regular ICU care even though we all did the best we could for the patients to survive and receive relatively safe care (#47).”*

The diluted competency and heavy workload forced the prioritization of essential life-saving measures, which entailed trade-offs with regard to ordinary care activities, deprioritized administration, such as documentation, reporting adverse events and ensuring the flow of updated information, and showed a decreased focus on personal wellbeing.

### 3.4. Intervening Contextual Conditions 

***Microlevel leadership and coworkership*** associated with great commitment and responsibility among front-line staff and ICU management was perceived to be the main enabler of the escalation of intensive care capacity. One manager’s narrative expressed this situation as follows:

*“I could sense a tremendous loyalty within the organization that made the impossible possible. I’m really impressed with how quickly the organization was put together; the employees just fixed it! (#23).”*

The crisis led to a joint focus on managing the situation. Preexisting routines and clinical operation protocols provided a solid base for performance, and competent ordinary staff facilitated the supervision of temporary staff. The professionals also noted that they were used to manage emergencies and collaborate in temporary teams, thus facilitating adaptation, flexibility, innovative solutions and collegial support. The continuity of coworkers and responsiveness to individuals’ different competences were perceived to facilitate collaboration. A nonhierarchical team climate promoted bottom-up initiatives but also led to difficulties with regard to articulating and accepting a chain of command, which sometimes aggravated distinct decisions.

***Available premises, as well as material and human resources,*** affected the demand–capacity balance and the ability to adapt. Large premises with access to oxygen and compressed air, as well as the possibility of isolation, facilitated the establishment of temporary ICUs with large cohorts. An existing patient record system that enabled information access from a distance facilitated rapid assessment and provided an overview of the situation. Scarce spare medical equipment, and shortages in crisis stockpiles of pharmaceuticals and consumables, along with growing global demands, restricted the availability of material resources. Additionally, the participants highlighted that the availability of human resources was limited by the prepandemic staffing situation (at the border with regard to ordinary operational commitment) and a national lack of intensive care competency.

***Mesolevel organizational culture and adaptations*** affected the ways in which ICU management could manage the situation. The organizational culture implied a trusting mesolevel leadership, which allowed ICU management to exercise great autonomy at the microlevel. Regional crisis management was established. However, the response at the mesolevel and macrolevel was initially perceived as insufficient, which caused actions to be taken at the microlevel. As described in a narrative related by one manager,

*“In general, you can say that the response in our region was driven by the operational level. It was mainly ideas and initiatives from the Department of Anesthesia and Intensive Care and the Department of Infectious Diseases that created the action plans that formed the basis of the region’s way of facing the pandemic. Many decisions were made locally, anchored with partners and realized before senior management was informed of what was happening. From my side, there is both a frustration that the management did not act and a relief that what we said at the operational level was listened to (#68).”*

Collaboration among departments and hospitals in the region was facilitated by a sense of emergency. The general desire to contribute caused ordinary bureaucracy and economic constraints to be set aside, which enabled people to develop rapid solutions to emerging issues. The redistribution of staff was facilitated by prioritizations sanctioned at the mesolevel, which led to the suspension of nonimperative care and other commitments. Stricter ICU admission criteria were enabled by new intermediate units to care for patients who were in need of advanced care without mechanical ventilation. Additionally, early discharge from the ICU was facilitated by new step-down units for rehabilitation. ICU management was also provided with administrative support from the HR and IT departments. Practical support was provided by the infection control department, pharmacists and the service department to support supply and cleaning. Mental health support and economic compensation for staff working with COVID-19 were also provided; these measures were appreciated but perceived to be insufficient.

***Macrolevel organization and adaptations*** also affected intensive care. For example, policies regarding restrictions, PPE and ethical priorities were provided by national authorities. Dissemination of knowledge was facilitated by professional associations and informal networks. Prior to the pandemic, there was no national coordination of intensive care, which impeded the provision of initial support. As one physician concluded during an interview,

*“It was quite late that the door to other hospitals was opened among our intensive care units in the country… If you have had a different mindset, that we are together in this, Sweden is ONE intensive care clinic… Then, I think, we would have managed better (#28).”*

As a result of diverse national disease dissemination and informal contacts, the transportation of patients to less strained regions became possible, and the burdened regions could be supported through the provision of voluntary intensive care competencies. Over time, national ICU capacity was monitored, and transportation was coordinated and organized. While this was appreciated, a lack of understanding regarding local qualifications occasionally led to a suboptimal choice of ICU for individual patients

***Demands and support from civil society and international affairs*** that affected intensive care included the inflow of patients, as well as support from trade unions, spiritual leaders, family and friends, media reports and support from the public. For example, the media reported on the staff members as heroes, and the public organized food distribution to staff, all of which strengthened their fighting spirit. However, family demands and worries sometimes added to the perceived strain, and some restrictions in society also aggravated the possibility of commuting to work using public transportation. Additionally, international affairs affected the ICU. For example, competition and barriers to transportation and export obstructed purchases of essential equipment and consumables. On the other hand, lessons learned and evidence found in other countries were disseminated and utilized.

### 3.5. Processual Consequences

The processual consequences arose from the adaptations implemented. Working conditions influenced both the quality of care and patient safety, and vice versa. Additionally, both aspects were influenced by and contributed to managing commitment and learning. This situation was described by a registered nurse in a narrative as follows:

*“It was terrible! The room was not adapted for ICU care. I had never been to the ward and didn’t know the premises; the staff who would help were not used to intensive care, so I had to supervise them as well, even though I had two really ill ICU patients… This day was absolutely not safe for the patients! (#45).”*

***Managing commitment and learning over time*** was indicated as, despite the threatening chaos, a basic level of intensive care was provided, and it was never necessary to implement the emergency ethical priority policy from the National Board of Health and Welfare. When the operational leadership of intensive care was transferred to a regional coordinator, a helicopter perspective was created; patient flow and overall care directives were perceived as efficient, while a lack of these functions led to disorganization and fluctuating care. The reorganization of ICU management and regional crisis management was considered to be successful because it enabled overall control and because alterations and measures could be rapidly developed and implemented.

The compilation of unambiguous information and daily meetings with the management facilitated compliance with alterations, while inconsistent information and lack of meetings led to insecurity. Over time, knowledge of the disease also increased, which led to improved treatment and less insecurity. Intensive care competence was successively disseminated to other professionals, the experience of temporary staff members increased, roles were settled, and patient care became routine. Demand– capacity imbalances were mitigated by increased resources, the decreasing inflow of patients and organizational learning. One interviewed physician described this situation as follows:

*“It’s been an incredible journey that you were not prepared for. But that it worked, sort of. It’s really cool (#26).”*

***Diluted competence, impaired quality of care and patient safety*** were related to demand–capacity imbalances and forced trade-offs. The lack of ICU competency on the part of temporary staff entailed that, under the supervision of a few ordinary staff members, patients received care from staff without specialist competence. This combination of diluted competence, high workload and strained staff limited time with patients and partly forced preventive care to be set aside, which was perceived to dehumanize the patients. The provision of care based on evidence concerning other diseases, and insufficient forums for the exchange of experiences, led to some disagreements regarding treatment strategies. Overall, this situation led to difficulties in making appropriate individual plans for patients, which affected the continuity and quality of care.

Substitute pharmaceuticals and equipment entailed unnecessary deep sedation and partly suboptimal ventilation, which prolonged the time spent on mechanical ventilation. This situation was perceived to put further strain on the ICU capacity and to affect patient outcomes negatively. The staff were forced to manage medical equipment for which they were not trained, and stretched margins of consumables also led to risks to patient safety. Initially, PPE with long sleeves and crowded premises led to the dissemination of hospital-acquired infections. Less frequent position changes and suboptimal mattresses led to pressure ulcers that were typically avoidable. The prioritization of patients with COVID-19 who were in need of invasive mechanical ventilation was also perceived to impact care quality for other patients negatively. One physician described this situation in a narrative as follows:

*“What has been tough is that we have not had general ICU beds, which I think has meant that patients with higher monitoring needs have ended up in a regular ward or that we have had to transport unstable patients unnecessarily (#35).”*

The transportation of critically ill patients exposed them to increased risks. On the other hand, the professionals noted that without those trade-offs, some patients would have been rejected from intensive care with even more catastrophic consequences for patient safety.

***Impaired work environment and working conditions*** were related to high demands in relation to control of the situation on the part of both management and staff. Temporary large ICUs were utilized without safety inspections. These premises were physically stressful environments that featured high levels of noise, heat and crowding. A suboptimal design created difficulties in reaching medical equipment and material. On the other hand, the large cohort enabled resource allocation and the surveillance of patients, as well as collegial support and the dissemination of knowledge.

Continuously changing demands and adaptations, work schedules that were changed at short notice, new colleagues and altered roles and responsibilities were perceived as stressful and contributed to staff feeling like game pieces. PPE entailed both security and physical discomfort and impaired people’s ability to interact and communicate with patients and colleagues. The deficiency of PPE, the use of replacement products and frequently altered recommendations created distrust, and concern with being sick and disseminating that sickness to loved ones. The redistribution of staff placed administrative burdens on first-line managers, which limited their ability to support staff. Overall, impaired working conditions affected the social lives and wellbeing of both staff and managers.

### 3.6. Aftermath

As the first wave of COVID-19 faded away, the aftermath of the escalation process was discernible. This ambiguous experience was summarized in a narrative by a registered nurse as follows:

*“It’s like an experience that I would have liked to have avoided in a way, but now that it’s here, I don’t want to be without it…How we did in March-April, we didn’t do at all in May-June. We did it differently, treated differently; we learned a lot… At that time, you were so up to it in some way, high on adrenaline or what should I say. Then, when you got a vacation, which we actually got for the summer, the air went out of you. And the air hasn’t really returned yet… So, there are thoughts about both the present and the future and how people will cope (#80).”*

***Individual and organizational development*** was indicated by many of the involved staff, who perceived personal development and noted that they were proud of what had been accomplished. Compared to the prepandemic situation, the value of external staff, such as physiotherapists, clinical pharmacists and cleaners, was perceived to have increased, and previous organizational barriers were overcome. Additionally, the adaptations that the crisis necessitated also entailed the rapid and successful implementation of improvements and efficiency increases that had been discussed but not implemented before the pandemic. Some types of measures that had previously rarely been performed, such as placing the patient in a prone position, became routine and performed with less concern.

After the first pandemic wave, knowledge of the future development and prognosis of the pandemic was still scarce, but lessons had been learned regarding the dissemination, treatments and prognosis of the disease. The establishment of local stockpiles of consumables and pharmaceuticals was initiated. Insights into necessary considerations, including those related to crisis management and information dissemination, were obtained. Compared to the prepandemic level, preparedness for the outbreak of potential diseases was perceived to have improved. The pandemic was also perceived to elicit a general focus on and understanding of the importance of intensive care, which facilitated recruitment and led to the allocation of resources for permanently increased ICU capacity.

***Pent-up care and staff with recovery needs*** were indicated by the tremendous backlogs that resulted from the postponement of nonimperative care. Hence, many patients were waiting to undergo surgery, assessments and other treatments, which caused the staff to worry. The adaptations implemented to manage the escalation of intensive care were also accomplished at the expense of exhausted and sometimes traumatized staff. Despite the need for recovery, they were required to manage the pent-up care needs for other groups of patients, which left them no time for relaxation. Additionally, uncertainty regarding eventual new outbreaks of the disease and disappointing staff policies regarding redistribution, schedules, support and financial compensation impaired their faith in the future.

### 3.7. Recommendaions Regarding How to Optimize the Prerequisites for Resilient Performance 

Based on the participants’ experiences of the escalation process, 41 recommendations regarding how to optimize the prerequisites for resilient performance were identified (see Table 3). As described in the subcategories above, some of the prerequisites were perceived to be present in the studied settings, while others were perceived improvement opportunities. 

## 4. Discussion

The conceptual model presented in this research describes and explains the escalation process of intensive care during the first wave of the COVID-19 pandemic as a transition from threatening chaos to temporary order through a complex process of adaptation. Although the EPIC model was generated inductively from empirical data, it resonates with empirical experiences in other settings and different aspects of the resilience and surge capacity frameworks suggested below. The model, and the recommendations provided, contribute to our understanding of the notion that adaptations must be viewed as a complex process [1,16,28,33] and that resilient performance is affected by the interdependencies between different levels of the system in the organizational context [10,11].

### 4.1. Relations between the EPIC Model and Existing Frameworks

The cornerstones of resilient performance are the capacities to anticipate, monitor, respond and learn [8]. The emerging situation was partly anticipated by gradual insights into the need for preparation, but this situation also led to unanticipated, urgent and massive needs that triggered the escalation in ICU capacity. A situation of multifactorial strain [24,25,27], caused by this escalation, created altered demands and uncertainty, which were the main concern of all parties involved in the escalation of intensive care. This situation could also be described in terms of a set of demand–capacity misalignments [13] related to the four Ss [24,25] with respect to the extensive need for room and infection control (space), demand–capacity imbalances regarding material and human resources (stuff and staff), rapidly altered care conditions and increased demands on governance and communication (system). Additionally, the novelty of the situation and the extensive need to obtain and disseminate knowledge could be related to a fifth S, namely ´science´ [24].

To maintain the service’s operational commitment and core values [27], i.e., to provide high-quality care [2], both proactive and reactive responses [14,26] occurred, which could be related to the four potentials to anticipate, monitor, respond and learn [8]. These responses included multiple adaptations, which reflected a situated and structural resilient performance at different levels of the system throughout the escalation process, e.g., on different spatial and temporal scales [13,14]. Initially, many microlevel-initiated ad hoc solutions were implemented, but gradually, through monitoring and learning [8,13,27], the ability to anticipate the situation and implement more proactive responses increased at all levels.

This response led to positive as well as negative processual consequences, thus confirming that adaptations are not normative [13,15,16,17,27]. Despite the threatening chaos, intensive care could be provided to patients in need, and it was never necessary to implement the emergency ethical priority directive from the National Board of Health and Welfare [34]. However, the adaptations that were essential for maintaining commitment also led to additional strain, which forced further responses and trade-offs in an onward iterative complex process of adaptation, which also affected working conditions, the work environment, the quality of care and patient safety. 

As shown (Figure 2), all components in the EPIC model were contextually interrelated, and the adaptive capacity was facilitated and challenged by intervening conditions at different levels of the system, thus confirming the interdependencies of a CAS [3,16]. The process of adaptation continued until the contagion faded away and the aftermath of the escalation was discernible. As in other settings [4,35,36], pent-up care and staff with recovery needs were prominent. However, individual and organizational development (commonly referred to as learning or growth [8,27]) were also visible, thus possibly improving preparedness for future challenges. 

### 4.2. The Complex Process of Adaptation

In line with the EPIC model, scholars have viewed resilience as a process rather than as an end state; in this context, adaptive strategies are distributed in combination rather than in a linear manner [1,28,33]. To ensure organizational support [16], adaptation should be viewed as a complex process that features a high degree of interrelatedness among various actors and levels of the system. Therefore, the incorporation of complexity is proposed to refine our current understanding of resilience in healthcare [1].

Intensive care capacity in the regions under study increased by 491% and 215%, respectively [21]. Despite this massive escalation, capacity was saturated, an issue which was solved by interhospital collaboration, as in other countries [5,37]. Despite global experiences with crises related to disease and surge capacity recommendations, the preparedness of intensive care for a crisis of the magnitude of the COVID-19 pandemic was insufficient [6,24]. Metaphorically, the rails were built as the train sped along.

The main enabler of the massive escalation of care capacity was revealed to be great commitment on the part of individuals, including their ability to take the initiative and their willingness to take on expanded responsibilities, which was also emphasized by others [22,38]. Furthermore, the dominant motive underlying such commitment was altruistic [38], as individuals were both driven by a sense of emergency and encouraged by support from civil society: “If not me, who could solve the situation?” Initially, insufficient response at the mesolevel and the macrolevel led to bottom-up initiatives and self-organization at the microlevel, which is a common invoked strength of a CAS [10,11,12]. Competence and experiences derived from temporary team collaborations and emergency situations facilitated adaptation, flexibility, the development of innovative solutions and support, thus confirming the findings of Ambrose and colleagues [38].

Other essential enablers of this escalation, which have been highlighted numerous times [19,37,39], were the collaboration and redistribution of resources. Lundberg and Johansson [27] describe collaboration in the context of a crisis in terms of a fluctuating resilience value network. Moving resources (resource-pooling) among different nodes in the network can create stability in a CAS that is under pressure. Resource-pooling in the context of this escalation was enabled by deprioritizing other commitments, initially at the local level within each department and gradually also among various departments and hospitals. Before formalized collaboration was established, informal networks enabled the dissemination of knowledge as well as interhospital collaboration.

Collaboration and networking are based on trust, communication and the willingness to take on roles and responsibilities that are outside one’s comfort zone [27], which in the present study was facilitated by a collective sense of emergency; this point has also been emphasized by experiences in other settings, such as in Italy [39] and the US [37,38]. However, information dissemination was one of the main challenges, and as in other settings [38], this issue was mitigated only partly through established communication strategies.

The establishment of temporary ICUs to provide cohort care was enabled by large premises with access to oxygen, compressed air and isolation facilities, as described by other researchers [5,6,36,37]. These temporary ICUs facilitated capacity escalation but entailed suboptimal working conditions and challenges for patient safety, points which have also been emphasized by previous researchers [6,22,36]. To meet future escalation needs in a more expedient manner, the development of preprepared “silent” ICUs, which can be used for other activities during normal circumstances and easily activated in a surge response. is suggested [6]. Additionally, it was necessary to equip and staff these temporary ICUs. When the inflow of critically ill patients is high, specialist competence is crucial for the ability to adapt and escalate hospital-care capacity and for leading temporary or junior colleagues [19]. The challenges posed by working conditions and staffing hospital beds were already an important issue before the pandemic [40,41], and a reason to activate contingency plans [42]. The redistribution of staff [5,27] based on priorities and sanctions at the mesolevel enabled the increased number of ICU beds to be staffed. However, in line with previous findings [4], the need for external staff, intrahospital staff movement, overtime and new schedules were major challenges that led to frustration among individuals. The number of redistributed staff diluted intensive care competence and caused ordinary staff members to experience additional strain. Additionally, as in other settings [22,38], to manage these acutely altered demands and compensate for insufficient material recourses, front-line staff were required to adapt their practice. Due to these rapid alterations, a large proportion of the response was a reaction to immediate needs; however, a proactive response also existed in terms of preparing in advance based on the anticipated circumstances. Existing guidelines and procedures, as well as the competence of ordinary staff, provided a solid foundation for the supervision of temporary staff, thus highlighting the importance of cherishing continuity. Lessons learned from our study and other publications [5,6,25,43] include the importance of the specialist competences possessed by intensive care professionals and the need for local stockpiles of consumables and pharmaceuticals.

In line with subsequently published guidelines for surge capacity [24,25], the ICU management adjusted plans, negotiated and prioritized different stakeholders’ interests, reorganized and tried to compensate for deficiencies by adapting the ICU’s capacity in terms of space, stuff, staff, system and science. Although corresponding types of surge response have been described by other researchers [5,6,36,37,38,44,45], in light of possible publication bias, the bottom-up approach seems to distinguish Sweden from other countries. Self-organization and microlevel autonomy were facilitated by trusting leadership, which has been recognized as an enabler of adaptive capacity [17,27]; subsequently, these outcomes were also supported by crisis management, as well as administrative and practical support, from actors at the mesolevel. Microlevel autonomy was perceived as empowering and as an enabler of opportune adaptations; however, it also led to uncertainty and frustration. In line with these findings, resilient healthcare in a crisis requires a combination of bottom-up and top-down approaches [46]. However, governance and leadership, as well as front-line adaptations in the pandemic, require further exploration.

### 4.3. Adaptive Capacity as Both an Enabler and a Challenge within a CAS

In a CAS, adaptive capacity may be both an enabler of high-quality care and a challenge for organizations and healthcare workers [13,15,16,17,27]. As visualized in the EPIC model, this point was especially prominent during the pandemic. However, adaptations in everyday practice can also be problematic and, for example, include a disparity between escalation policies (work-as-imagined) and how escalation processes are managed in practice (work-as-done) [47]. Adaptation encompasses the ability in a CAS to self-organize, reconcile conflicting goals, reevaluate priorities, innovate and cope with external demands [12]. However, it entails the risk of reducing resilient performance to the level of individuals´ adaptability, which in turn can mask system deficiencies and organizational weaknesses and be misinterpreted as the adaptive capacity of the system. In the present settings, resilient performance greatly relied on individuals’ capacity to adapt to challenges, which is a fragile safety strategy.

Crises can force the negotiation of core values [27]. In the escalation of intensive care, to avoid the chaos threatened by the pandemic, ICU management made adaptations and trade-offs at the microlevel, which indeed enabled care to be provided but also led to unfavorable processual consequences, which in turn resulted in front-line adaptations and trade-offs. However, individuals’ degrees of freedom and ability to adapt have limits [48]. High demands for control in this situation led to impaired working conditions and work environments for both managers and front-line staff, thus risking exhaustion and sick leave [4,35,48]. Although some have reported total success [44], staff working in different settings attest to the related impacts on the quality of care [19,22,46]. Accordingly, the motivation to continue working in healthcare can be lost, leading to the immediate consequence of a shortage of competence.

Rasmussen’s system safety model [48] highlights the facts that a system under pressure can increase the demand for efficiency, and that individuals under pressure may reduce their activities to maintain their effectiveness. Both gradients thereby drive the system toward the margin of what can be considered safe [48]. Hence, a holistic consideration of the working conditions and wellbeing of staff members is important from both individual and system perspectives [6,49]. The system safety model [48] could be beneficial for visualizing a state and facilitating discussions regarding the room for action in a crisis. However, the precise location of the tipping point with respect to what a system or an individual can manage before experiencing exhaustion or leading to patient harm is difficult to anticipate. 

### 4.4. Clinical Implications

A CAS is difficult to predict under normal conditions, and this complexity was exacerbated by the pandemic. On a global scale, this situation should make us humble in light of what healthcare systems, and the professionals who work for them, were capable of in the struggle to provide high-quality care during this extreme crisis.

Based on the escalation of intensive care as an empirical case, the EPIC model (Figure 2) highlights this complexity by illuminating the interrelatedness of multiple components, and the iterative nature of the process of adaptation. To our knowledge, this complexity has not previously been explained on the basis of empirical data in the context of a healthcare crisis. Based on the findings, resilient performance can be operationalized, and intervening conditions and consequences can be explained. Additionally, recommendations are provided for the micro-meso-macrolevels regarding how to optimize the prerequisites for resilient performance in both standard situations and future crises in intensive care (see Table 3). Hence, the lessons drawn from the findings can guide strategies for improving resilient performance in healthcare in both ICUs and other healthcare settings. However, to deepen our understanding of resilience in healthcare during a further crisis, future research should explore other perspectives and other contexts in the healthcare system. This work is in progress.

### 4.5. Methodological Considerations 

This study had certain limitations that should be noted. First, ICUs in only two Swedish regions were included, and none of these ICUs were associated with university hospitals. However, these regions were of average size nationally and heavily affected by the pandemic; they thus offered lessons to be learned. The transferability of the findings is enhanced by in-depth contextual descriptions. Second, the sampling was based on voluntary registration through a broadly spread invitation, which did not enable us to conduct a nonresponse analysis. Data were collected from staff through written narratives and interviews; thus, the quality of data was affected by the informants’ ability to express themselves, and the risk of recall bias cannot be disregarded. However, data were collected in close connection to the first wave of the pandemic, and theoretical saturation was achieved.

Research in the field of resilience in healthcare has been criticized for being insufficiently grounded in empirical data, which risks the uncritical adoption of concepts drawn from other disciplines based on what is assumed rather than what is demonstrated [28]. To our knowledge, this research is the first study to use grounded theory [30] to study resilience in the escalation of intensive care during a healthcare crisis. To describe and explain phenomena that occur in the complex context of healthcare inevitability entails simplifications. Complex interrelationships are to some extent presented linearly. However, simplified models can be useful for generating and spreading insights into complex phenomena, for which purpose grounded theory was a feasible approach.

## 5. Conclusions

The present study focused on describing and explaining the escalation process of intensive care during the initial wave of COVID-19 from a microlevel organizational perspective in a comprehensible manner, while embracing the interdependencies with the meso and macro levels. Involving all levels of the system, this escalation was conceptualized as a transition from threatening chaos to temporary order through a complex process of adaptation. To ensure that healthcare and ICUs are prepared for future crises, the components of space, stuff, staff, system and science, with associated continuity plans, must be implemented, anchored and communicated to actors at all levels of the system.

This novel empirically grounded EPIC model contributes to current knowledge by offering an in-depth understanding of what occurs during the process of adaptation; in other words, it explains how resilient performance was expressed, what worked and why, and with what consequences. This understanding is applicable to both clinical practice and theoretical development within the field of resilience in healthcare.

## Figures and Tables

**Table 1:** Participant characteristics.

	Gender		Mean Age (Min–Max)	Region		Data Source		Total Number
	Female	Male		A	B	Written	Interview	
Assistant nurses	6	-	49 (36–58)	3	3	4	2	6
Registered nurses ^1^	28	9	48 (28–62)	22	15	30	7	37
Physicians ^2^	5	11	50 (32–69)	12	4	5	11	16
Managers ^3^	10	1	50 (45–64)	5	6	8	3	11
Total	49	21	48 (28–69)	42	28	47	23	70

^1^ Including nurses specialized in intensive care and nurses with other or no specialist training. ^2^ Including anesthesiologists, residents and interns. ^3^ Including first-line managers (head of care unit) and second-line managers (head of department) within the departments that operated the ICUs (microlevel managers).

**Table 2:** The core categories, main categories and subcategories in the conceptual model of the escalation process of intensive care.

Core Category	Main Categories	Subcategories
From threatening chaos to temporary order through a complex process of adaptation	Triggers	A novel contagious disease
		Gradual insights into the need for preparation
		Urgent need for increased ICU capacity
		Decreased inflow enabling de-escalation
	Altered demands and uncertainty	Rapidly altered care conditions
		Extensive need for room and infection control
		Demand–capacity imbalance regarding material and human resources
		Increased demands on governance, collaboration and communication
	Proactive and reactive responses	Preparing and planning to the best of one’s ability
		Reorganizing the management and information structure
		Prioritizing and reorganizing patient flow
		Restructuring and compensating premises and material resources
		Redistributing staff and adjusting roles
		Alterations and trade-offs in patient care
	Intervening contextual conditions	Microlevel leadership and coworkership
		Available premises, material and human recourses
		Mesolevel organizational culture and adaptations
		Macrolevel organization and adaptations
		Demands and support from civil society and international affairs
	Processual consequences	Managing commitment and learning over time
		Diluted competence, impaired quality of care and patient safety
		Impaired work environment and working conditions
	Aftermath	Individual and organizational development
		Pent-up care and staff with recovery needs

**Table 3:** Recommendations at the micro-, meso- and macrosystem levels regarding how to optimize the prerequisites for resilient performance in intensive care ^1^.

System Level	Component	Recommendations
Micro-level	Space	Sufficient permanent intensive care capacity
	Stuff	Designated staff for repetitive inventories of medical equipment, supplies and pharmaceutical items
	Staff	Retention of experienced intensive care professionals by valuing their competence and commitment Opportunity for reflection and support for staff (group reflection/individual support) Service staff to unload work from healthcare professionals An implemented plan, including sufficient training, for roles and responsibilities in the context of a surge continuum (conventional, contingency and crisis care), including front-line leadership and a plan for sanctioned trade-offs The retention of developed competencies on the part of temporary staff through, e.g., rotational positions/training Scheduled training and simulations Staffing high-volume, high-intensity ICUs with 24/7 coverage of physicians with specialist competence
	System	Distributed leadership that values and empowers front-line decisions Consensus among management regarding core value and goals Information and a communication structure that ensure accurate and timely information and the participation of staff from all categories Alternative forms of contact with patient families when visiting is restricted (through telephone or video calls) A prepared local crisis management/incident command for tactical and operational leadership
		An electronic patient record system that enables access from a distance
	Science	Clear routines and clinical operation protocols The provision of updated information in a compiled (web-based) document in a crisis situation Structures for searching and implementing new evidence as well as phasing out outdated methods The retention and permanent use of improvements that were implemented during the crisis
Meso-level	Space	A flexible floor-plan with the possibility of temporary co-locating many patients in large premises Preprepared “silent” ICUs that can be used for other activities during normal circumstances and can easily be activated during a crisis The provision of premises for training and simulations (e.g., clinical training centers)
	Stuff	Local stockpiles of consumables and drugs
	Staff	Expedient IT support for staffing Optimize human recourses by ensuring continuity and utilizing the right competence in the right place The provision of psychological crisis support
	System	The establishment of value networks for collaboration across organizational boarders (both among departments/hospitals in the same region and across regions) Continuity plans for long-term crises, which are anchored and communicated to actors at all levels of the system Valuing and empowering microlevel competence and decisions Designated communication and daily updates at a specified place and time during a crisis with the possibility of asking questions A reduction in the burden of administration on first-line to enable them to have sufficient time to provide leadership and staff support Prepared mesolevel crisis management/incident command for tactical and strategic leadership
	Science	The provision of support for literature searches Support from Department of infection prevention and control
Macro-level	Space	-
	Stuff	Common procurement and contingency warehouses for medical equipment, consumables (incl. PPE) and drugs
	Staff	A long-term overall plan to assure current and future staffing and different specialist competencies for intensive care Financial and recreation compensation for staff facing strain
	System	Coordination of intensive care capacity, human and material resources Consensus criteria for the focus and level of care, including criteria for intensive care and do-not-resuscitate decisions A supportive society
	Science	National and international networks for communicating information regarding new diseases and evidence concerning treatment rapidly

^1^ Based on lessons learned from the findings of the escalation process of intensive care.

## Data Availability

The datasets used and analyzed in the current study are available from the corresponding author upon reasonable request.
